# Supplementary material for: Activation of Downstream mTORC1 Target Ribosomal Protein S6 Kinase (S6K) Can Be Found in a Subgroup of Dutch Patients with Granulomatous Pulmonary Disease
Source: Cells. 2021 Dec 15;10(12):3545. doi: 10.3390/cells10123545 (PMC8700352; doi:10.3390/cells10123545)
Supplement: Supplementary file 1 [file cells-10-03545-s001.zip › cells-1508130-Supplementary.pdf]

cells MDPI

**Table S1. Characteristics of sarcoidosis patients**. In total, granuloma specimens from lung (31 samples), lymph nodes (22 samples), skin (17 samples), bone marrow (2 samples), nose (one sample), and salivary gland (one sample) have been stained. Data is shown as absolute numbers and percentage. COS: Clinical outcome status, SFN: Small fiber neuropathy, Scadding stages: 0 = Normal chest radiograph; I = Bilateral hilar lymphadenopathy (BHL); II = BHL with pulmonary infiltrates; III = pulmonary infiltrates without BHL; IV = fibrosis

| Parameter | | n | |
|---|---|---|---|
| Age [a] (years) | | 74 | 44.90 ± 12.47 |
| Male sex | | 41/74 | 55.4% |
| Ever smoker | | 41/74 | 55.4% |
| Caucasian | | 62/74 | 83.8% |
| Löfgren syndrome | | 2/74 | 2.7% |
| Medication at time of biopsy | | 7/74 | 9.5% |
| Third-line therapy [b] | | 26/74 | 35.1% |
| COS group 2 years follow up | | 70/74 | |
| Group A | | 22 | 31.4% |
| Group B | | 48 | 68.6% |
| COS group 5 years follow up | | 45/74 | |
| Group A | | 13 | 28.9% |
| Group B | | 32 | 71.1% |
| Scadding stage at time biopsy | | 71/74 | |
| Stage 0 | | 5 | 7.0% |
| Stage 1 | | 17 | 23.9% |
| Stage 2 | | 27 | 38.0% |
| Stage 3 | | 12 | 16.9% |
| Stage 4 | | 10 | 14.1% |
| Inflammatory biomarkers [c] | *Ref.range* | | |
| ACE (U/L) | *12–70* | 23/74 | 56.32 ± 35.29 |
| sIL-2R (pg/mL) | *<3000* | 17/74 | 5980.82 ± 3958.10 |
| Involved organs | | | |
| Lung | | 31/74 | 41.9% |
| Lymph nodes | | 23/74 | 31.1% |
| Skin | | 18/74 | 24.3% |
| Eyes | | 6/74 | 8.1% |
| Liver | | 8/74 | 10.8% |
| Heart | | 12/74 | 16.2% |
| Spleen | | 3/74 | 4.1% |
| Bones | | 4/74 | 5.4% |
| Nerve system | | 13/74 | 17.6% |
| *SFN* | | 9/74 | 12.2% |

[a] Age at time of biopsy and is shown as mean ± SD. [b] Third-line therapy consisted of infliximab use at follow up. [c] biomarkers at time of diagnosis and is shown as mean ± SD.

**Table S2. Characteristics of patients with other granulomatous disorders**. In total 29 patients with HP, 7 patients with GPA, 1 patients with EGPA, 1 patient with tuberculosis and 1 patient with pneumoconiosis were included. Granuloma specimens from lung (35 samples), lymph nodes (2 samples) and nose (2 samples) have been stained. Data is shown as absolute numbers and percentage.

| Parameter | n | |
|---|---|---|
| Age [a] (years) | 39 | 52.40 ± 11.47 |
| Male sex | 21/39 | 53.9% |
| Ever smoker | 22/39 | 56.4% |
| Caucasian | 38/39 | 97.4% |
| Medication [a] | 5/39 | 12.8% |
| Inducing agent HP | | |
| Unknown | 14/29 | 48.3% |
| Birds | 12/29 | 41.4% |
| Farmer's lung | 3/29 | 10.3% |

[a] Age and medication at time of biopsy and is shown as mean ± SD.
